# Nomogram Predicting Renal Insufficiency after Nephroureterectomy for Upper Tract Urothelial Carcinoma in the Chinese Population: Exclusion of Ineligible Candidates for Adjuvant Chemotherapy

**DOI:** 10.1155/2014/529186

**Published:** 2014-08-10

**Authors:** Dong Fang, Qifu Zhang, Xuesong Li, Cheng Qian, Gengyan Xiong, Lei Zhang, Xiaopeng Chen, Xiaoyu Zhang, Wei Yu, Zhisong He, Liqun Zhou

**Affiliations:** ^1^Department of Urology, Peking University First Hospital, Institute of Urology, Peking University, National Urological Cancer Centre, No. 8 Xishiku Street, Xicheng District, Beijing 100034, China; ^2^Department of Urology, Jilin Tumour Hospital, No. 1018 Huguang Road, Chaoyang District, Changchun, Jilin 130021, China; ^3^Department of Urology, Tangdu Hospital, Fourth Military Medical University, No.1 Xinsi Road, Baqiao District, Xi'an, Shaanxi 710038, China

## Abstract

*Objectives.* To report the decline of renal function after radical nephroureterectomy (RNU) in upper tract urothelial carcinoma (UTUC) patients and to develop a nomogram to predict ineligibility for cisplatin-based adjuvant chemotherapy (AC). *Methods.* We retrospectively analyzed 606 consecutive Chinese UTUC patients treated by RNU from 2000 to 2010. We chose an eGFR of 60 and 45 ml/min/1.73 m^2^ as cut-offs for full-dose and reduced-dose AC eligibility. *Results.* Median eGFR for all patients before and after surgery was 64 and 49 ml/min/1.73 m^2^ (*P* < 0.001). The proportion of patients ineligible to receive full-dose and reduced-dose AC changed from 42% to 74% and from 20% to 38.1%. Older age (OR = 1.007), preoperative eGFR (OR = 0.993), absence of hydronephrosis (OR = 0.801), smaller tumor size (OR = 0.962), and tumor without multifocality (OR = 0.876) were predictive for ineligibility for full-dose AC. Preoperative eGFR (OR = 0.991), absence of hydronephrosis (OR = 0.881), tumor located in renal pelvis (OR = 1.164), and smaller tumor size (OR = 0.969) could predict ineligibility for reduced-dose AC. The c-index of the two models was 0.757 and 0.836. Postoperative renal function was not associated with worse survival. *Conclusions.* Older age, lower preoperative eGFR, smaller tumor size, tumor located in renal pelvis, and absence of hydronephrosis or multifocality were predictors of postoperative renal insufficiency.

## 1. Introduction

Although radical nephroureterectomy (RNU) with excision of the bladder cuff is the gold-standard treatment for upper tract urothelial carcinomas (UTUC) [1], the oncologic outcomes for patients with high-grade or non-organ-confined disease remain poor, with 5-year cancer-specific survival rates less than 60% [2–4]. Multimodal approaches have been suggested and perioperative chemotherapy has been considered as an option to improve disease control [5–7].

The use of cisplatin-based chemotherapy has gained greater acceptance as evidenced by its use in neoadjuvant and adjuvant therapy, especially in patients with pT3-4 or pTxN+ [5–8]. But cisplatin-based therapy was associated with a higher risk of severe nephrotoxicity, and creatine clearance was important in determining whether patients should be treated with cisplatin [9]. However, the present limited ability to predict tumor stage and grade accurately before surgery makes it difficult to select proper candidates for neoadjuvant therapy, while the loss of renal unit would limit the use of cisplatin-based chemotherapy in adjuvant therapy [10, 11].

Several reports have evaluated changes in renal function following RNU and demonstrated that the decline in renal function may render a substantial number of patients ineligible to receive adjuvant chemotherapy (AC) [10–15]. The ability to predict which patients would develop renal insufficiency following RNU would be extremely useful. Previous studies provided scarce information on risk stratification for worse chronic kidney disease (CKD) after RNU [13–15], and there are few published reports from research centers in China.

Therefore, in this large single-center cohort of patients, we sought to reveal the prevalence of CKD before and after RNU and to develop a nomogram to predict ineligibility for AC, which would help to accurately predict postoperative renal function and thus provide more optimal and personalized risk-based therapy options. Besides, we evaluated the association between postoperative renal function and survival.

## 2. Materials and Methods

### 2.1. Patient Selection

Following institutional review board approval and written informed consent from patients, we initially collected the clinicopathological data of 912 consecutive UTUC patients who received treatment in the Department of Urology, Peking University First Hospital, from 2000 to 2012. This was a large cohort drawing from 27 different provinces or autonomous regions of China. Patients underwent nephron-sparing surgery instead of RNU, with bilateral synchronous UTUC, and previous histories for UTUC, incomplete data on pre- or postoperative serum creatine (Scr), or no follow-up data were excluded. Six hundred and six patients were finally enrolled for evaluation.

All patients were diagnosed using computed tomography (CT) or magnetic resonance imaging (MRI), urologic ultrasound, and in some patients ureteroscopy with or without biopsy. All patients underwent surgery within two months after the occurrence of symptoms. Lower ureter and bladder cuff excision was performed through the Gibson incision in all cases. None of these patients received neoadjuvant chemotherapy (NC), while, for several patients, adjuvant chemotherapy or radiotherapy was administered when evidence of distant metastasis or retroperitoneal recurrence was documented on condition that patients had good general condition.

### 2.2. Patients Evaluation

The estimated glomerular filtration rate (eGFR) was calculated using the modified glomerular filtration rate estimating equation for Chinese patients: eGFR (mL/min/1.73 m^2^) = 175 × Scr^−1.234^ × age^−0.179^ (× 0.79 if female) [16]. Comparison of eGFR before and after surgery was performed using the Scr drawn closest to 7 days after surgery (range: 3 days to 1 month after surgery). This timing was selected to best approximate the measured serum creatinine that would reflect the direct effect of RNU on renal function. And most patients could be included with available pre- and postoperative Scr data. We chose an eGFR of 60 and 45 mL/min/1.73 m^2^ as possible cut-offs for full-dose and reduced-dose cisplatin-based AC. The eGFR cut-off of 45 mL/min/1.73 m^2^ was defined for its compromise between lower limits for reduced-dose cisplatin in previous studies [17–19]; besides, it was cited as a more strict definition of CKD [12].

All pathological specimens were re-reviewed by a dedicated genitourinary pathologist to confirm the reproducibility of the diagnosis. Tumor stage was assessed according to the 2002 Union for International Cancer Control (UICC) TNM classification of malignant tumors. Tumor grading was assessed according to the World Health Organization (WHO) classification of 1973. Tumor architecture was defined as papillary or sessile by the examination of the final specimen. Tumor location was divided into 2 areas (renal pelvis and ureter) based on the site of the dominant lesion. Tumor multifocality was defined as the synchronous presence of two or more pathologically confirmed macroscopic tumors in any location. Ipsilateral hydronephrosis (HN) was determined by MRI or CT before operation.

### 2.3. Follow-Up Schedule

For patients who were followed up at our institute, the follow-up regimen of the affected patients included cystoscopy every 3 months for the first 3 years. The cystoscopy intervals were extended to 1 year thereafter. Chest X-ray, urine cytology, Scr, and abdominal ultrasound or CT/MRI were examined at the same time. The cause of death was determined by the patients' treating physicians or by death certificates. Follow-ups were censored until their last visit or death.

### 2.4. Statistical Analysis

Pearson's test and chi-square test were used to test the distribution of categorical variables, and the Mann-Whitney *U* test and paired-sample *t*-test were used for continuous variables. Multivariate logistic regression was used to calculate the predictive factors. Only variables that were identified as significant by the univariate analysis were considered for the multivariate analysis. Log-rank test was used in survival analysis. Multivariable logistic regression coefficients were used to generate a nomogram for impaired renal function [20]. Discrimination was measured using Harrell's concordance index (c-index), which is similar to the area under the receiver operating characteristic curve. Calibration was measured by calibration plots, which were generated to explore the nomograms performance using 200 bootstrap resamples.

The generation of the nomogram and calibration plots was performed with the R open-source statistical software, and other statistical tests were performed with SPSS 20.0 (IBM Corp., Armonk, NY, USA). All reported *P* values were single-sided with statistical significance considered at *P* < 0.05.

## 3. Results and Discussion

### 3.1. Patients Characteristics and Changes in Renal Function

The median patient age was 69 years (range: 25–94 years). The distribution of UTUC pathological stage in this cohort was pTa, pT1, pT2, pT3, and pT4 in 20 (3.3%) patients, 189 (31.2%) patients, 213 (35.1%) patients, 173 (28.5%) patients, and 11 (1.8%) patients, respectively. Pathological grades 1–3 tumors were present in 18 (3.0%) individuals, 333 (55.0%) individuals, and 255 (42.1%) individuals, respectively.

Median eGFR for all patients prior to surgery was 64 (interquartile range, IQR 49–81) mL/min/1.73 m^2^, while after RNU it was 49 (IQR 38–60) mL/min/1.73 m^2^. Comparison of pre- and postoperative eGFR revealed a mean decrease of 15 mL/min/1.73 m^2^ and a median percentage loss of 24% after RNU (*P* < 0.001).

**Figure 1:**
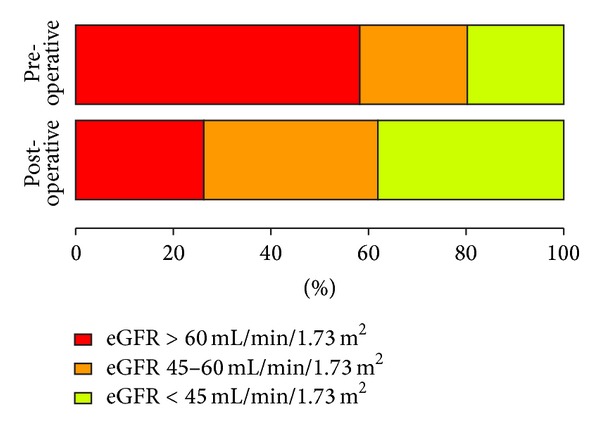
Percentage of patients with pre- and postoperative eGFR in selected ranges (over 60 mL/min/1.73 m^2^, between 45 and 60 mL/min/1.73 m^2^, and lower than 45 mL/min/1.73 m^2^).

Using 60 mL/min/1.73 m^2^ as the eligibility cut-off for full-dose AC, 58% of the study population was eligible before surgery, whereas only 26% remained eligible following RNU. Using a cut-off of 45 mL/min/1.73 m^2^ for reduced-dose AC, 80% was eligible preoperatively, whereas only 61.9% remained above this cut-off after surgery (Figure 1).

Only 11 patients (1.8%) in this cohort actually received AC at our institute, with mean preoperative eGFR 71.5 mL/min/1.73 m^2^ and postoperative eGFR 54.5 mL/min/1.73 m^2^.

### 3.2. Risk Factors for Impaired Renal Function

**Figure 2:**
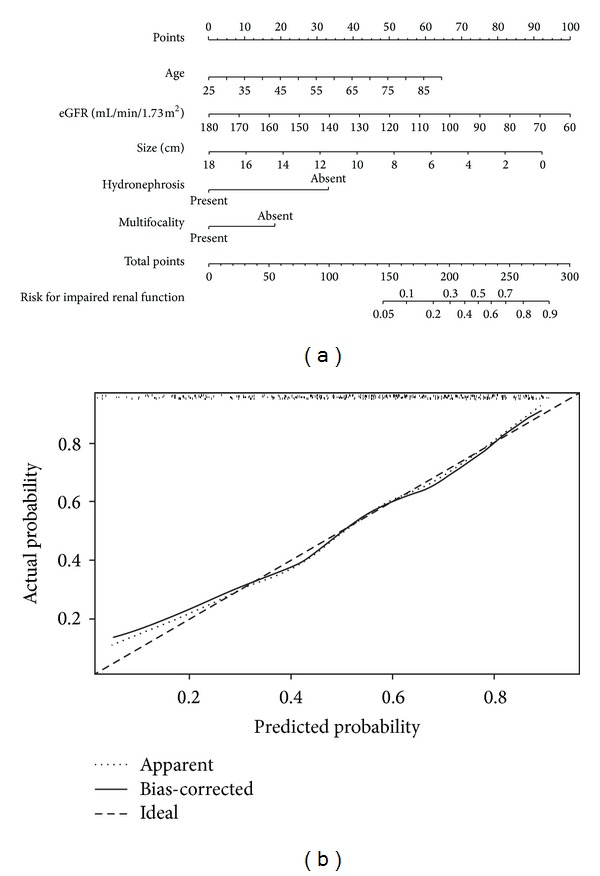
Nomogram (a) and calibration plot (b) for prediction of ineligibility to receive full-dose adjuvant chemotherapy with a c-index of 0.757.

Of the 353 possible candidates for NC, 193 (54.7%) were judged ineligible to undergo AC with their postoperative eGFR below 60 mL/min/1.73 m^2^. On univariate analysis, older age (*P* = 0.001), lower preoperative eGFR (*P* = 0.003), tumor located in renal pelvis (*P* = 0.007), absence of preoperative HN (*P* < 0.001), tumor without multifocality (*P* = 0.006), tumor size (*P* = 0.001), lower tumor stage (*P* = 0.001), and papillary architecture (*P* < 0.001) were associated with postoperative eGFR lower than 60 mL/min/1.73 m^2^. Multivariate analysis controlling for all preoperative factors demonstrated that older age (OR = 1.007 per year), lower preoperative eGFR (OR = 0.993 per mL/min/1.73 m^2^), absence of preoperative HN (OR = 0.801), smaller tumor size (OR = 0.962 per centimeter), and tumor without multifocality (OR = 0.876) were independent risk factors predicting ineligibility for full-dose AC (Table 1). We used logistic regression coefficients to generate a corresponding nomogram (Figure 2(a)) and calibration plot (Figure 2(b)). The accuracy of the model nomogram measured by c-index was 0.757.

**Figure 3:**
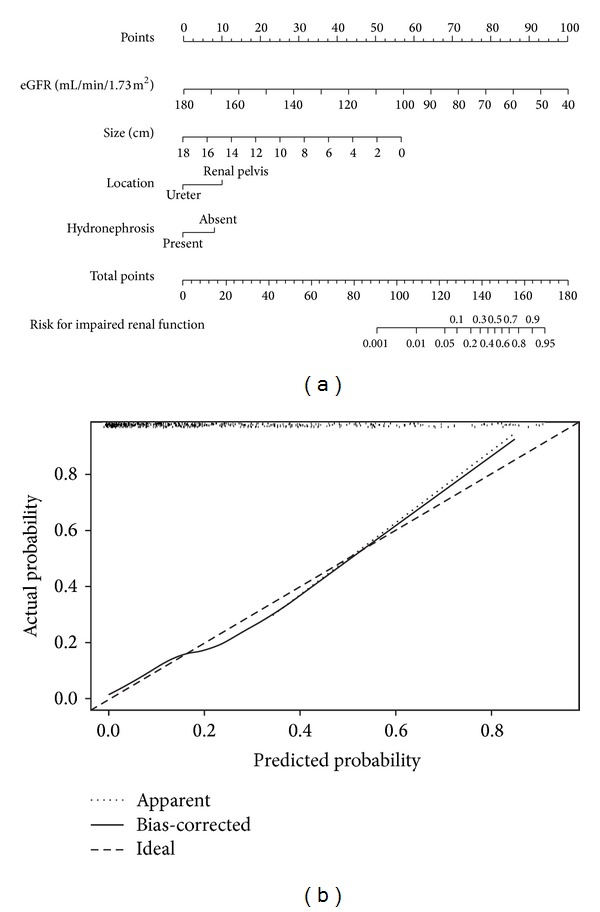
Nomogram (a) and calibration plot (b) for prediction of ineligibility to receive reduced-dose adjuvant chemotherapy with a c-index of 0.836.

Similarly, when we defined cut-off of 45 mL/min/1.73 m^2^, preoperative eGFR (OR = 0.991 per mL/min/1.73 m^2^), absence of preoperative HN (OR = 0.881), tumor located in renal pelvis (OR = 1.164), and smaller tumor size (OR = 0.969) independently predicted impaired postoperative renal function in multivariate analysis (Table 2). The corresponding nomogram and calibration plot were shown in Figure 3. The accuracy of the model nomogram measured by c-index was 0.836.

### 3.3. Predictive Role on Survival

**Figure 4:**
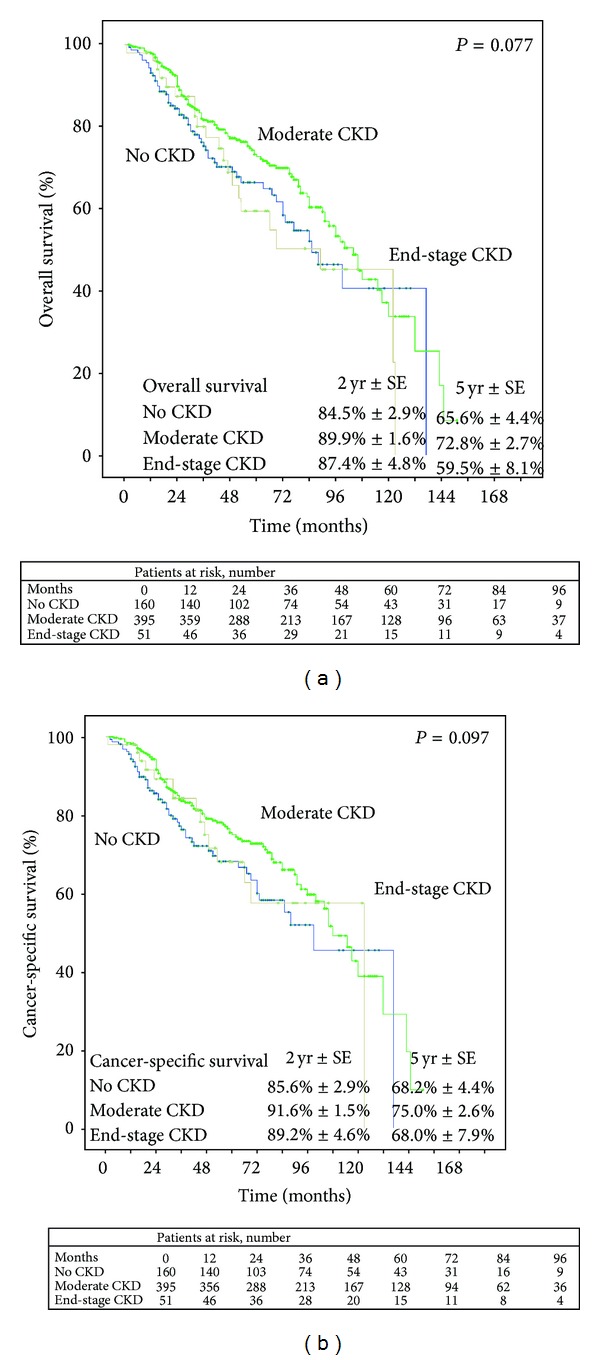
Estimated Kaplan-Meier overall survival curves (a) (*P* = 0.077) and cancer-specific survival curves (b) (*P* = 0.097) stratified by postoperative renal function.

The median follow-up duration of this cohort of patients was 56 (IQR 24–72) months. One hundred and ninety-three patients (31.2%) died, and 166 patients (27.4%) died of urothelial cancer. The 5-year overall survival and cancer-specific survival were 69.8% and 72.6%, respectively. By log-rank test, postoperative renal function was not associated with worse OS (*P* = 0.077) or worse CSS (*P* = 0.097) (Figure 4). Besides, use of AC demonstrated no effect on survival (data not shown).

### 3.4. Discussion

The result of the present research confirmed that eGFR deteriorates significantly following RNU, and a substantial proportion (over 30%) of patients would miss the opportunity to undergo AC for impaired renal function, similar to previous reports [10–12]. Even when we set cut-off at 45 mL/min/1.73 m^2^ for reduced dose of cisplatin-based chemotherapy, we could notice that although 80% of patients were qualified for NC, RNU rendered nearly 20% of patients ineligible for AC. Besides, we found no association between postoperative CKD and survival, which is in accordance with previous studies [12]. Though theoretically CKD is related to higher risk of cardiovascular events, the association of eGFR with survival in UTUC patients has not been determined and needs to be further clarified.

Due to the decline of eGFR, previous studies suggest strong consideration of neoadjuvant regimens when chemotherapy is indicated [10, 12]. But the current staging modalities hindered the extensive use of NC. As the only commonly accepted measure to get biopsy before RNU, ureteroscopy is associated with perioperative complications and higher risk of intravesical recurrence [21]. Without biopsy, we cannot firmly exclude the possibility of another pathological diagnosis instead of transitional cell carcinoma. Besides, predictive models for non-organ-confined or high-grade disease by preoperative factors in previous studies have not been testified in population-based study [22–25]. A casual NC might result in overtreatment in low-risk disease. On the other hand, AC could be carried after pathological examination of final specimen. Thus, prediction of postoperative renal function is important to detect patients not suitable for AC.

Older age and preoperative eGFR were proved to be predictive of decline of eGFR in previous reports [12, 15, 26]. There was no consensus about the predictive role of other clinical factors. Hoshino et al. [14] found the absence of higher grade HN was independent risk factor for patients ineligible for AC, while results were contrary in Rodriguez Faba's research [15]. No explanation for these results was provided in these studies. A probable hypothesis for our results is that the presence of HN was always accompanied by thinner renal cortex and reduced eGFR. Total renal function would be compensated by the contralateral kidney. Thus, the resection of the impaired kidney would not result in significant decline of total eGFR as it only takes a small proportion. For patients with same preoperative eGFR, those with ipsilateral HN would probably have a better contralateral kidney and, as a result, a probable higher postoperative eGFR. Similarly, although not evidenced in previous clinical trials, it is easy to deduce that large tumor size, multifocality, and ureteral location would be associated with impaired function of the kidney with tumor (the kidney that would be removed); thus, they were demonstrated to be “protective” factor for postoperative eGFR after adjusting for preoperative renal function.

It is a pity that in most patients split renal function studies by nuclear renal scans were not routinely obtained before surgery. Evidence is scarce but, considering its function in evaluating the preoperative eGFR of the contralateral kidney directly, it might play an important role in predictive postoperative renal function after RNU.

Our model could help clinicians in optimizing timing of chemotherapy regimens and providing personalized therapy options based on preoperative factors. For patients evaluated as high risk for postoperative renal insufficiency, if systematic chemotherapy is considered, NC is recommended before their renal function is impaired, while, for patients less likely to suffer from decreased eGFR lower than 60 mL/min/1.73 m^2^, clinicians could carefully evaluate the necessity of NC. Unless strongly indicated (e.g., high grade in biopsy, suspicious of lymph node metastasis, and/or adjacent organs invaded), clinicians could perform RNU without delay and carry AC if required based on final pathology.

NC has been demonstrated to decrease tumor burden and improve patients' survival [8, 27], while the effect of AC on prognosis was unsatisfied [5–7, 28, 29]. However, many of these trials were retrospective, single-center study with small sample size, and the regimen as well as doses was limited by poor postoperative renal function and was not standardized. Although the previous results of NC are promising, we should not neglect the possible benefits of AC which clearly needs more research.

There are some limitations in our study, especially the limitation of retrospective itself, and our study cohort might have been subject to selection and recall bias. Additionally, since we focused on early time-points in postoperative Scr, we could not exclude the possibility of spuriously low Scr measurements due to perioperative intravenous hydration or spuriously high Scr measurements as it is too early for the contralateral kidney to fully compensate. But our result could be interpreted as evaluation of acute renal disease short-term after RNU, and Kaag et al. [13] demonstrated that the decline of renal function after RNU showed no evidence of recovery over time. Another limitation is that, due to various reasons, there were extremely few patients that actually received AC.

Despite these limitations, to our knowledge, it is the first research that provides an applicable tool to predict postoperative renal insufficiency that could help risk stratification and treatment strategies selection. Future studies on long-term monitoring of renal function and prospective clinical trials on AC would be required.

## 4. Conclusions

In the Chinese patients with UTUC, older age, lower preoperative eGFR, smaller tumor size, tumor located in renal pelvis, and absence of HN or tumor multifocality were demonstrated to be significant predictors of impaired renal function following RNU. The nomogram accurately predicts ineligibility for AC. Postoperative renal function did not correlate with patients' survival. The clinical significance of those results needs to be further assessed in external multi-institutional validation cohorts.

## Figures and Tables

**Table 1:** Predictive factors for identifying patients ineligible to receive full dose of cisplatin-based adjuvant chemotherapy^#^.

	Postoperative eGFR (mL/min/1.73 m^2^)			Univariate analysis		Multivariate analysis		
	All	≥60	<60	Chi-square	*P* value	OR	95% CI	*P* value
All, number (%)	353 (100)	160 (45.3)	193 (54.7)					
Gender, number (%)				0.004	0.516			
Male	167 (47.3)	76 (47.5)	91 (47.2)					
Female	186 (52.7)	84 (52.5)	102 (52.8)					
Age^∧^, number (%)				10.966	**0.001∗**	1.007	1.002–1.012	**0.002∗**
<70	200 (56.7)	106 (66.3)	94 (48.7)					
≥70	153 (43.3)	54 (33.8)	99 (51.3)					
Preoperative eGFR (mL/min/1.73 m^2^)^∧^, number (%)				8.235	**0.003∗**	0.993	0.991–0.997	**<0.001∗**
<90	87 (24.6)	51 (31.9)	36 (18.7)					
≥90	266 (75.4)	109 (68.1)	157 (81.3)					
Previous or concomitant BT, number (%)				0.145	0.417			
No	313 (88.7)	143 (89.4)	170 (88.1)					
Yes	40 (11.3)	17 (10.6)	23 (11.9)					
Side, number (%)				0.533	0.267			
Left	173 (49.0)	75 (46.9)	98 (50.8)					
Right	180 (51.0)	85 (53.1)	95 (49.2)					
Location, number (%)				6.513	**0.007∗**	1.071	0.955–1.202	0.240
Ureter	144 (40.8)	77 (48.1)	67 (34.7)					
Pelvis	209 (59.2)	83 (51.9)	126 (65.3)					
Hydronephrosis, number (%)				22.657	**<0.001∗**	0.801	0.714–0.899	**<0.001∗**
No	188 (53.3)	63 (39.4)	125 (64.8)					
Yes	165 (46.7)	97 (60.6)	68 (35.2)					
Multifocality, number (%)				7.038	**0.006∗**	0.876	0.771–0.996	**0.044∗**
No	294 (83.3)	124 (77.5)	170 (88.1)					
Yes	59 (16.7)	36 (22.5)	23 (11.9)					
DM, number (%)				0.980	0.200			
No	297 (84.1)	138 (86.3)	159 (82.4)					
Yes	56 (15.9)	22 (13.8)	34 (17.6)					
Hypertension, number (%)				2.931	0.055			
No	228 (64.6)	111 (69.4)	117 (60.6)					
Yes	125 (35.4)	49 (30.6)	76 (39.4)					
Smoking, number (%)				2.853	0.060			
No	281 (79.6)	121 (75.6)	160 (82.9)					
Yes	72 (20.4)	39 (24.4)	33 (17.1)					
Tumor size (cm)^∧^, number (%)				10.514	**0.001∗**	0.962	0.942–0.983	**<0.001∗**
<3	190 (53.8)	71 (44.4)	119 (61.7)					
≥3	163 (46.2)	89 (55.6)	74 (38.3)					
Architecture, number (%)				11.830	**<0.001∗**			
Papillary	268 (75.9)	109 (68.1)	159 (82.4)					
Sessile	80 (22.7)	50 (31.3)	30 (15.5)					
Stage, number (%)				10.873	**0.001∗**			
Ta/T1/T2	233 (66.0)	91 (56.9)	142 (73.6)					
T3/T4	120 (34.0)	69 (43.1)	51 (26.4)					
Grade, number (%)				2.829	0.058			
G1-2	209 (59.2)	87 (54.4)	122 (63.2)					
G3	144 (40.8)	73 (45.6)	71 (36.8)					
Tumor necrosis, number (%)				2.766	0.069			
No	319 (30.4)	140 (87.5)	179 (92.7)					
Yes	34 (9.6)	20 (12.5)	14 (7.3)					
CIS, number (%)				0.389	0.374			
Absent	342 (96.9)	154 (96.3)	188 (97.4)					
Present	11 (3.1)	6 (3.8)	5 (2.6)					

*Statistically significant.

^#^Only patients with preoperative eGFR ≥ 60 mL/min/1.73 m^2^ were included.

^∧^Initially calculated as binary variables in univariate analysis and used as linear variable in multivariate analysis.

OR: odds ratio; CI: confidence interval; eGFR: estimated glomerular filtration rate; BT: bladder tumor; DM: diabetes mellitus; CIS: carcinoma in situ.

**Table 2:** Predictive factors for identifying patients ineligible to receive reduced dose of cisplatin-based adjuvant chemotherapy^#^.

	Postoperative eGFR (mL/min/1.73 m^2^)			Univariate analysis		Multivariate analysis		
	All	≥45	<45	Chi-square	*P* value	OR	95% CI	*P* value
All, number (%)	485 (100)	375 (77.3)	110 (22.3)					
Gender, number (%)				0.685	0.236			
Male	224 (46.2)	177 (47.2)	47 (42.7)					
Female	261 (53.8)	198 (52.8)	63 (57.3)					
Age^∧^, number (%)				6.773	**0.006∗**	0.999	0.996–1.003	0.922
<70	260 (53.6)	213 (56.8)	47 (42.7)					
≥70	225 (46.4)	162 (43.2)	63 (57.3)					
Preoperative eGFR (mL/min/1.73 m^2^)^∧^, number (%)				34.365	**<0.001∗**	0.991	0.989–0.993	**<0.001∗**
<60	353 (72.8)	297 (79.2)	56 (50.9)					
≥60	132 (27.2)	78 (20.8)	54 (49.1)					
Previous or concomitant BT, number (%)				0.004	0.546			
No	418 (86.2)	323 (86.1)	95 (86.4)					
Yes	67 (13.8)	52 (13.9)	15 (13.6)					
Side, number (%)				0.645	0.244			
Left	235 (48.5)	178 (47.5)	57 (51.8)					
Right	250 (51.5)	197 (52.5)	53 (48.2)					
Location, number (%)				9.325	**0.002∗**	1.164	1.074–1.262	**<0.001∗**
Ureter	234 (48.2)	195 (52.0)	39 (35.5)					
Pelvis	251 (51.8)	180 (48.0)	71 (64.5)					
Hydronephrosis, number (%)				12.523	**<0.001∗**	0.881	0.813–0.956	**0.002∗**
No	228 (47.0)	160 (42.7)	68 (61.8)					
Yes	257 (53.0)	215 (57.3)	42 (38.2)					
Multifocality, number (%)				0.853	0.215			
No	398 (82.1)	311 (82.9)	87 (79.1)					
Yes	87 (17.9)	64 (17.1)	23 (20.9)					
DM, number (%)				0.091	0.444			
No	401 (92.7)	309 (82.4)	92 (83.6)					
Yes	84 (17.3)	66 (17.6)	18 (16.4)					
Hypertension, number (%)				0.877	0.204			
No	292 (60.2)	230 (61.3)	62 (56.4)					
Yes	193 (39.8)	145 (38.7)	48 (43.6)					
Smoking, number (%)				0.276	0.344			
No	392 (80.8)	305 (81.3)	87 (79.1)					
Yes	93 (19.2)	70 (18.7)	23 (20.9)					
Tumor size (cm)^∧^, number (%)				16.163	**<0.001∗**	0.969	0.955–0.983	**0.001∗**
<3	267 (55.1)	188 (50.1)	79 (71.8)					
≥3	218 (44.9)	187 (49.9)	31 (28.2)					
Architecture, number (%)				13.392	**<0.001∗**			
Papillary	362 (74.6)	266 (70.9)	96 (87.3)					
Sessile	117 (24.1)	105 (28.0)	12 (10.9)					
Stage, number (%)				19.838	**<0.001∗**			
Ta/T1/T2	330 (68.0)	236 (62.9)	94 (85.5)					
T3/T4	155 (32.0)	139 (37.1)	16 (14.5)					
Grade, number (%)				9.525	**0.001∗**			
G1-2	282 (58.1)	204 (54.4)	78 (70.9)					
G3	203 (41.9)	171 (45.6)	32 (29.1)					
Tumor necrosis, number (%)				3.385	**0.043∗**			
No	436 (89.9)	332 (88.5)	104 (94.5)					
Yes	49 (10.1)	43 (11.5)	6 (5.5)					
CIS, number (%)				0.579	0.350			
Absent	471 (97.1)	363 (96.8)	108 (98.2)					
Present	14 (2.9)	12 (3.2)	2 (1.8)					

*Statistically significant.

^#^Only patients with preoperative eGFR ≥ 45 mL/min/1.73 m^2^ were included.

^∧^Initially calculated as binary variables in univariate analysis and used as linear variable in multivariate analysis.

OR: odds ratio; CI: confidence interval; eGFR: estimated glomerular filtration rate; BT: bladder tumor; DM: diabetes mellitus; CIS: carcinoma in situ.
